# The micro-RNA expression profile predicts the severity of SARS-CoV-2 infection

**DOI:** 10.1038/s41598-025-01229-2

**Published:** 2025-05-17

**Authors:** Ewa Pius-Sadowska, Piotr Kulig, Anna Niedźwiedź, Bartłomiej Baumert, Dorota Rogińska, Karolina Łuczkowska, Anna Sobuś, Miłosz Parczewski, Miłosz Kawa, Edyta Paczkowska, Bogusław Machaliński

**Affiliations:** 1 Department of General Pathology, Pomeranian Medical University in Szczecin, Al. Powstańców Wielkopolskich 72, 70-111 Szczecin, Poland; 2 Department of Infectious, Tropical Diseases and Immune Deficiency, Pomeranian Medical University in Szczecin, Arkońska 4 Street, 71-455 Szczecin, Poland

**Keywords:** COVID-19, MiRNA, Epigenetic, Severe COVID-19, SARS-CoV-2 infection, Infectious diseases, Viral infection

## Abstract

Although much is known about the pathophysiology of severe COVID-19, there are still areas that remain to be determined. It is well established that the pivotal molecular event is a hyperinflammatory response also referred to as a cytokine storm. The aim of this retrospective cohort study was to determine miRNAs which might be predictive for the admission to the intensive care unit (ICU). We analyzed blood samples from 210 COVID-19 patients and the control group consisted of 80 healthy individuals. Results revealed the miRNA expression pattern has the potential to predict the severity of COVID-19, reflecting the clinical symptoms of the infection, such as the need for oxygen therapy and concomitant pneumonia. In particular, low expression of miRNAs miR106a-5p, miR17-5p, miR181a-5p, miR191-5p, miR20a-5p and miR451a, especially in the initial phase of the disease, is associated with an unfavorable clinical course of SARS-CoV-2 infection (admission to the ICU).

## Introduction

The pathophysiology of the severe course of the COVID-19 has been investigated in detail due to its clinical significance and the large number of individuals affected by the disease. SARS-CoV-2 infection, because of its high contagiousness, prevalence and, in some cases, extremely severe clinical course, posed a huge challenge to health care systems around the world. Although majority of COVID-19 individuals experience mild to moderate infection, approximately 10%^1^ of cases suffer severe illness requiring admission to the intensive care unit (ICU). Moreover, severe COVID-19 can be fatal in certain circumstances, mainly in vulnerable populations^2^. It has been established that the key event influencing adverse clinical outcome is the hyperinflammatory response to the virus, often called the cytokine storm^3,4^. In this respect, the key mediators are pro-inflammatory cytokines, whose plasma concentrations are clearly increased^5^. Considering the results of studies on the pathophysiology of the severe course of the COVID-19, it can be concluded that it is associated with an increase in the plasma concentrations of various pro-inflammatory mediators. Therefore, the preceding molecular event must be an increase in the expression of the genes encoding the mentioned molecules. The rapid and global change in gene expression implies the involvement of epigenetic mechanisms that facilitate transcription or those acting later, at the post-transcriptional level.

The role of pro-inflammatory cytokines in the severe course of COVID-19 is very well established. However, much less is known about the underlying mechanisms that modulate gene expression of molecules mediating severe disease. Epigenetic is a subdiscipline of genetics that deals with the regulation of gene expression regardless of alterations in the nucleotide sequence. The main epigenetic mechanisms include DNA methylation, histone modifications, chromatin remodeling, and non-coding RNA^6^. Aberrant methylation profile has been linked to COVID-19 and disease severity. According to the study conducted by Bradic et al., alterations in the methylome of genes controlling the immune response (with particular emphasis on type I interferon signaling) and apoptotic pathways are associated with acute respiratory distress syndrome (ARDS) and adequately predict clinical outcome^7^. Furthermore, global changes in the epigenome, which manifest themselves particularly in alterations of immune-related signaling pathways, predict disease progression and patient prognosis^8^. Moreover, and of particular relevance in the context of these studies, the miRNA expression profile may be associated with disease severity and has been shown to have predictive value^9^.

Based on the above premises and the experience from our previous research, we defined the following aims of this study: (i) to establish the differences in the miRNA expression profile between mild infection and patients requiring ICU admission; (ii) to define putative epigenetic predictors of severe COVID-19 disease and non-favorable clinical outcome.

## Materials and methods

### Study group

This retrospective cohort study included 210 patients (50 ICU and 160 non-ICU patients) diagnosed with COVID-19 at the Department of Infectious, Tropical Diseases and Acquired Immunodeficiency at the Pomeranian Medical University in Szczecin, Poland. Nasopharyngeal swabs analyzed by real-time polymerase chain reaction (RT-PCR) were used to confirm SARS-CoV-2 virus infection. The control population comprised 80 healthy individuals from the hospital staff whose RT–PCR results for SARS-CoV-2 from nasopharyngeal swab were negative, and their ELISA results for SARS-CoV-2-specific IgG, IgM, and IgA antibodies were also negative. In the study design, ICU patients constituted the research group, while non-ICU individuals constituted the control group. The control population was included to validate the laboratory kits and ensure that SARS-CoV-2 patients had truly positive results. In study participants, blood was collected to assess the expression of predetermined genes, as well as the levels of specific chemokines and complement components. In addition, participants were required to complete comprehensive questionnaires regarding their overall health. The study was approved by the Ethics Committee of the Pomeranian Medical University in Szczecin (KB-0012/83/2020) and was in accordance with the principles of the Declaration of Helsinki. Before starting the study, each participant signed an informed consent form. Patients were enrolled to the study from July 2020 to April 2021. More details can be found in our previous articles^10,11^.

### General health questionnaire

All patients were interviewed and examined to document symptoms such as fever, shortness of breath, cough, cold, sore throat, fatigue, chest pain, changes in smell/taste, headache, body aches, and diarrhea, along with details of severity and duration of symptoms. Medical records were reviewed to collect data on laboratory test results, need for oxygen or respiratory support, presence of pneumonia on chest computed tomography, need for hemodialysis, and patient outcomes, including mortality. Additionally, patient demographics, family medical history, and pre-existing health conditions such as hypertension, hyperlipidemia, smoking, diabetes, cardiovascular problems, liver disease, respiratory disorders, rheumatic diseases, and previous cerebrovascular events were documented. The severity of COVID-19 cases was assessed retrospectively by categorizing COVID-19-infected patients into two groups according to the severity of the disease. Group 1 consisted of intensive care unit patients (ICU patients) requiring intensive care due to respiratory failure, hospital stays longer than 14 days due to COVID-19 or with a fatal outcome. Group 2 encompassed non-ICU patients, including asymptomatic or mildly symptomatic cases with an oxygen saturation level of at least 95%, not requiring hospitalization, as well as symptomatic patients, with an oxygen saturation below 95%, requiring hospitalization for up to fourteen days. More details can be found in our previous articles^10,11^.

### Material

#### Plasma collection

Peripheral blood samples were collected upon the patient’s admission to hospital on day 1, and subsequently during hospitalization/isolation on days 7, 14 and 28 following the COVID-19 diagnosis. Approximately 7.5 mL of peripheral blood was collected into EDTA tubes and centrifuged at 2000 rpm for 10 min. The resulting plasma was then transferred to a new tube and subjected to a second centrifugation cycle under the same parameters. Blood samples were collected only at the hospital, no samples were collected prior to admission.

#### Viral RNA isolation

Viral RNA isolation was performed using the MagMAX Viral/Pathogen II Nucleic Acid Isolation Kit (Thermo Fisher Scientific, ON, CA) according to the manufacturer’s protocol. MagMAX Viral/Pathogen nucleic acid isolation was processed using an automated KingFisher Flex instrument (Thermo Fisher Scientific, ON, CA). The isolation procedure has been described in detail in our previous articles^10,11^.

#### qRT–PCR assays for detecting SARS-CoV-2 RNA

For the detection of SARS-CoV-2 RNA, qRT-PCR assays were conducted utilizing a QuantStudio 5 PCR system and a TaqPath COVID-19 CE IVD RT-PCR Kit (Thermo Fisher Scientific, Markham, ON, Canada) following the manufacturer’s guidelines. After completion of RT-PCR, results were assessed using Applied Biosystems COVID-19 Interpretive Software v1.5.1 (Thermo Fisher Scientific, Markham, ON, Canada). Positive test results were determined if a minimum of 2 out of the 3 analyzed SARS-CoV-2 genes (ORF1ab, N, S) exhibited Ct values of ≤ 37. For further information, please refer to our prior publications^10,11^.

#### miRNA isolation

miRNA was extracted from 900 µL of plasma utilizing the NucleoSpin miRNA Plasma Mini kit designed for circulating miRNA (Macherey-Nagel, Düren, Germany) following the manufacturer’s instructions. Subsequently, the concentration of isolated miRNA from plasma was assessed using Qubit 4 Fluorometer (Thermo Fisher Scientific, Waltham, MA, USA) in conjunction with the Qubit microRNA Assay Kit (Thermo Fisher Scientific, Waltham, MA, USA).

#### Affymetrix GeneChip miRNA microarray and data analysis

miRNA was extracted from the plasma of all patients, although miRNA microarrays were solely conducted on selected samples (four representative samples at each time points and control group) and pooled to generate one sample for subsequent experimental procedures. The study utilized Affymetrix miRNA 4.1 Array Stripes (Affymetrix, Santa Clara, CA, USA) with *n* = 3 technical replicates for each miRNA probe. The procedure commenced with a poly(A) tailing reaction followed by ligation of the biotinylated signal molecule to the target RNA. The sample was then hybridized on an Affymetrix miRNA 4.1 Array Strip (Affymetrix, Santa Clara, CA, USA). The final step involved the addition of streptavidin-PE followed by array scanning using the Affymetrix GeneAtlas system (Affymetrix, Santa Clara, CA, USA). Microarray data analysis was performed using Bioconductor^12^. The normalized data were integrated into the description file “pd.mirna.4.1”, including miRNA names, types and sequences. Differential expression was determined by applying linear models to microarray data, facilitated by the “limma” library^13^. Differentially up- and down-regulated miRNAs were graphically presented in volcano plots based on predefined cut-off criteria (fold change > abs. 2). Experimentally validated miRNA target genes were obtained from the miRTarBase database, focusing solely on targets associated with differentially expressed miRNAs. The target gene lists from each comparison were functionally annotated and clustered using DAVID (Database for Annotation, Visualization and Integrated Discovery)^14^. Differentially expressed miRNA target symbols were entered into DAVID using the Bioconductor library “RDAVIDWebService”^15^, matching the targets to the corresponding gene ontology (GO) terms.

#### qRT-PCR for validation of selected miRNA expression

The results of miRNA microarray studies facilitated the identification of specific miRNAs with altered expression levels in the plasma of severely and moderately ill COVID-19 patients compared to healthy controls. Then, the expression of selected miRNAs (miR-106a-5p, miR-17-5p, miR-181a-5p, miR-191-5p, miR-20a-5p, miR-423-5p, miR-451a) was assessed through qRT-PCR on samples from all patients.

Initially, miRNA reverse transcription was conducted using the qScript microRNA cDNA Synthesis Kit (Quanta Biosciences, Beverly, MA, USA), which contains all the necessary components for qRT-PCR. Then, primers for miRNA were custom designed by miRPrimer and purchased at the Laboratory of DNA Sequencing and Oligonucleotide Synthesis at the Institute of Biochemistry and Biophysics of the Polish Academy of Sciences in Warsaw, Poland. The qRT-PCR protocol included an initial 10-minute denaturation at 95 °C, denaturation at 95 °C for 15 s, annealing at 54–57 °C (depending on the primer) for 30 s, and extension at 30 °C for 60 s. The relative gene expression was quantified using the comparative Ct method (2ΔCt), where ΔCt = (Ct of miRNA) - (Ct of endogenous control). All PCR products were characterized by high specificity by determining the melting point at a transition rate of 0.1 °C/s. The qRT-PCR reaction mixture comprised 5 µL of PerfeCTa SYBR Green SuperMix (Bio-Rad Inc., Hercules, CA, USA), 1 µL of microRNA cDNA, 0.2 µL of microRNA-specific primer, 0.2 µL of PerfeCTa Universal PCR Primer, and 4.6 µL of nuclease-free water. Reactions were performed on a Bio-Rad CFX96 Real-Time PCR Detection System (Bio-Rad Inc., Hercules, CA, USA), with all miRNA expressions performed in two technical replicates.

#### Statistical analysis

Quantitative data were expressed as mean and standard deviation. Medical history and medications taken the study were expressed as percentages. The Mann–Whitney test was used in our analyses to compare quantitative parameters between groups. Fisher’s exact test was implemented to assess the differences between categorical variables. A p value of < 0.05 was considered statistically significant. All calculations were performed in RStudio version 1.2.1335. ROC analysis was performed in PQ Stat software.

## Results

### Characteristic of the study group

Table 1 presents clinical characteristics of the study group. Overall, both ICU and non-ICU groups are homogenous. There were no significant differences in comorbidities, although ICU patients tended to have higher BMI and were slightly older.

**Table 1:** Clinical characteristics of the study group. Statistically significant p values are shown in bold. *NSAIDs* non steroid anti-inflammatory drugs.

Parameter	SARS-CoV2 negative controls (*n* = 80)	SARS-CoV2 positive patients (*n* = 210)	*p*	
Age (mean ± SD)	56.27 ± 5.56	57.78 ± 14.16	0.0522	
Sex (male/female)	5/75	114/96	**< 0.001**	
Body-mass index (mean ± SD)	25.85 ± 4,74	29.17 ± 5.52	**< 0.001**	

Parameter		SARS-CoV2 positive patients non-ICU (*n* = 160)	SARS-CoV2 positive patients ICU (*n* = 50)	p
Percent of patient population (%)		76.19	23.81	
Sex (male/female)		85/74	28/22	1
Age (mean ± SD)		56.26 ± 14,53	62.64 ± 11,79	**0.008524**
Body-mass index (mean ± SD)		28.87 ± 5.55	30.44 ± 5.42	**0.042**
Medical history				
% of patients with a given parameter	Cancer	10.63	10	1
	Diabetes	20.63	20	1
	Hypercholesterolemia	14,38	14	1
	Hypertension	44.38	54	0.2537
	Ischemic heart disease	7.5	12	0.3836
	Liver disease	0.63	2	0.42
	Respiratory system disease	8.13	14	0.2672
	Rheumatic disease	9.38	10	1
	Other diseases	33.13	50	**0.04224**
	Tobacco use (previous/now)	43.13/5.63	30/0	**< 0.001/< 0.001**
Currently taken medications:				
% of patients with a given parameter	Drugs taken on permanent basis	63.75	46	**0.02956**
	Anticoagulants	7.5	6	1
	Antihypertensive drugs	47.5	46	0.8715
	Anti-asthmatic drugs	8.13	10	0.7721
	Cardiac drugs	7.5	14	0.166
	NSAIDs	12.5	16	0.4855
	Statins	15	22	0.2769
	Other drugs	41.25	38	0.7413

Table 2 presents the most typical clinical manifestation of COVID-19. It should be noted that ICU patients significantly more frequently experienced dyspnea (*p* = 0.03). Moreover, the ICU group had a more frequent fatal outcome (*p* < 0.001).

**Table 2:** Data on disease severity and the presence of COVID-19 symptoms in SARS-CoV-2-positive patients. Statistically significant p values are shown in bold.

Parameter		SARS-CoV2 positive patients non-ICU (*n* = 160)	SARS-CoV2 positive patients ICU (*n* = 50)	*p*
% of patients with a given parameter	Chest pain	26.88	16	0.28
	Cough	71.25	60	0.87
	Dyspnoea	41.88	50	**0.03**
	Diarrhoea	23.75	26	0.86
	Fever above 38 °C	65	56	0.52
	Headache	35.63	24	**0.02**
	Pneumonia	84.38	72	0.67
	Smell/taste disorders	37.5	16	**0.002**
	Death	0.625	28	**< 0.001**

### SARS-CoV-2 infection is associated with global MiRNA downregulation

**Fig. 1:**
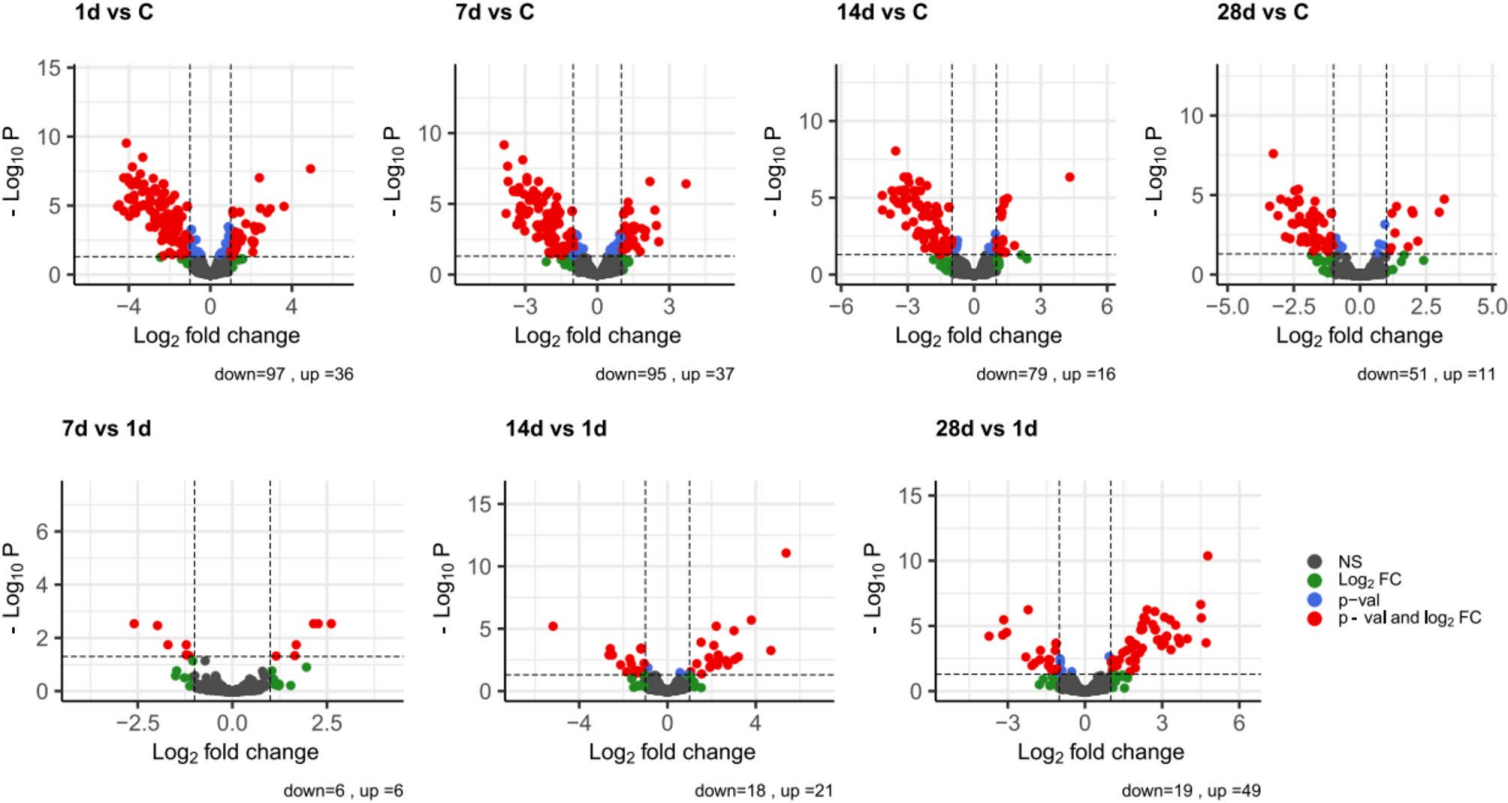
The volcano plot shows the total miRNAs expression profiles for a given pair of comparisons. Differentially expressed miRNAs are separated by gray dashed lines located at the cut-off values and were established according to the following parameters: fold change ≥ 2 and adjusted (adj.) p value < 0.05. Red dots represent miRNAs with a p-value < 0.05 and FC ≥ 2 and FC ≤ − 2; blue dots show miRNAs with p-value < 0.05 and FC ≤ 2 and FC ≥ − 2; green dots represent miRNAs with FC ≥ 2 and FC ≤ − 2, which do not present statistical significance; gray dots show miRNAs that did not meet either criterion. *FC* fold change.

SARS-CoV-2 infection is associated with global changes in miRNA expression (Fig. 1) between COVID-19 patients and healthy controls and within the study group between specific time points.

First, the analysis revealed an overall miRNA downregulation in COVID-19 individuals compared to control group on days 0 and 28.

Comparison within the study group (i.e., combined ICU and non-ICU patients) showed that the proportion of statistically significant upregulated and downregulated miRNAs was relatively the same, except for day 28, when twice as many upregulated miRNAs were identified compared to day 0. In summary, COVID-19 induced changes in miRNA expression that are most apparent at the beginning of infection (133 differently expressed miRNAs).

### Differences in miRNA expression profile in COVID-19 patients with mild symptoms compared to patients requiring admission to the intensive care unit

**Fig. 2:**
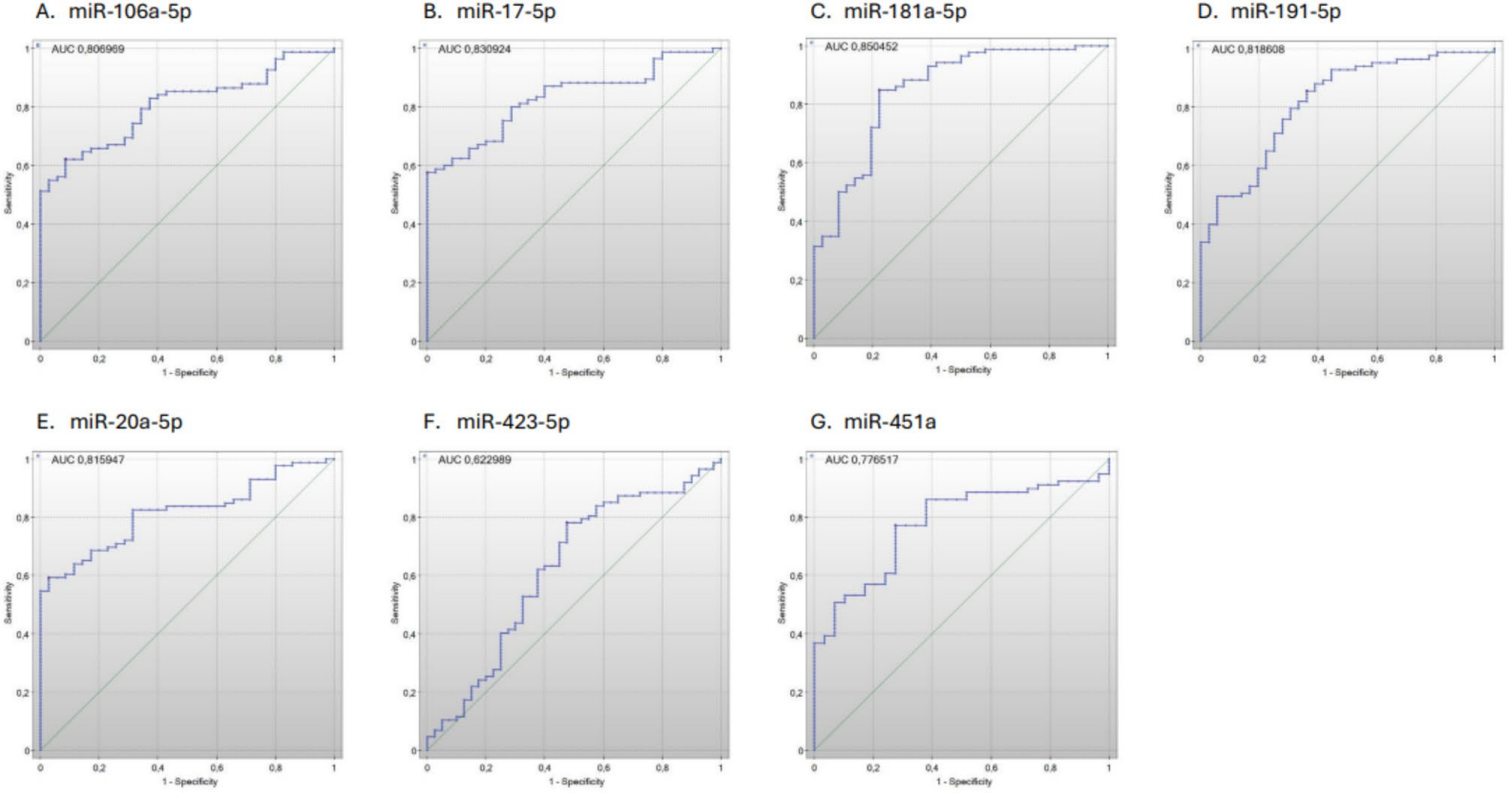
ROC curve analysis for the dysregulated miRNAs as predictive biomarkers for severe course of COVID-19. The ROC curves shown are representative of day 7 after SARS-CoV-2 infection.

In the next step, we selected the following miRNAs for further analysis (Table 3): miR106a-5p, miR17-5p, miR181a-5p, miR191-5p, miR20a-5p, miR423-5p and miR451a (Fig. 2). We performed Receiver Operating Characteristic (ROC) analysis. The Area Under the Curve (AUC) of ROC plots show the discriminatory ability of the biomarkers. Graphs present 1-specificity on the X axis and sensitivity on the Y axis. The maximum value for the AUC is 1.0 (which means 100% sensitive and 100% specific). ROC curves with an AUC > 0.7 are considered clinically useful. ROC analysis showed seven miRNAs with AUC > 0.7 (Table 3). Moreover, the indicated miRNAs had the highest fold change in expression. Additional criteria included the availability and quality of primers.

**Table 3:** ROC analysis results. In bold p-value < 0.05, which was considered statistically significant.

DeLong’s method				
miRNA	Time point	AUC	Cut-off point (Youden index method)	Two sided *p*-value
miR-106a-5p	1d	0.764763	> = 0.1122	**< 0.000001**
	7d	0.806969	> = 0.4186	**< 0**.**000001**
	14d	0.868481	> = 0.0649	**0**.**000004**
	28d	0.538265	> = 0.2270	0.647773
miR-17-5p	1d	0.760935	> = 0.0526	**0**.**000001**
	7d	0.830924	> = 0.9843	**< 0**.**000001**
	14d	0.830044	> = 0.1267	**0**.**000028**
	28d	0.531765	> = 0.0063	0.697249
miR-181a-5p	1d	0.806781	> = 0.1861	**< 0**.**000001**
	7d	0.850452	> = 0.5399	**< 0**.**000001**
	14d	0.811506	> = 0.1520	**0**.**000102**
	28d	0.577305	< = 0.5151	0.370347
miR-191-5p	1d	0.848637	> = 0.5391	**< 0**.**000001**
	7d	0.818608	> = 0.5190	**< 0**.**000001**
	14d	0.779268	> = 0.1752	**0**.**000434**
	28d	0.6075	> = 2.384017541	0.198169
miR-20a-5p	1d	0.700483	> = 0.1311	**0**.**000069**
	7d	0.815947	> = 1.5648	**< 0**.**000001**
	14d	0.81523	> = 0.1525	**0**.**000067**
	28d	0.609069	> = 0.42114	0.190594
miR-423-5p	1d	0.586041	> = 0.7530	0.068337
	7d	0.622989	> = 1.0303	**0**.**026319**
	14d	0.530612	> = 0.7811	0.68643
	28d	0.551471	< = 0.445938568	0.641969
miR-451a	1d	0.780964	> = 0.1693	**< 0**.**000001**
	7d	0.776517	> = 0.5665	**0**.**000011**
	14d	0.746032	> = 0.0657	**0**.**002144**
	28d	0.588889	> = 4.7954	0.3017

*AUC* area under curve.

First, we found that overall miRNA expression tends to be significantly lower in the ICU group compared with patients suffering from mild disease. Additionally, in both groups, with particular emphasis on individuals with an unfavorable clinical course (ICU group), miRNA expression tends to increase over time, which is particularly visible on the 28th day of the study.

Subsequently, our results revealed that the plasma concentrations of miR106a-5p, miR17-5p, miR181a-5p, miR191-5p, miR20a-5p, and miR451a miRNAs were significantly lower in the ICU group on days 0, 7, and 14, while it was not statistically significant differences in their expression at day 28, suggesting that miRNA expression increases over time in the ICU group, which may be attributed to the gradual resolution of the disease. The expression of miR423-5p miRNA was significantly higher in the non-ICU group only on day 7 suggesting that this miRNA is not strongly associated with ICU admission. On days 0, 14 and 28, no differences in its expression were found between the groups.

### MiRNA profile as a predictor of clinical symptoms and severity of SARS-CoV-2 infection

**Fig. 3:**
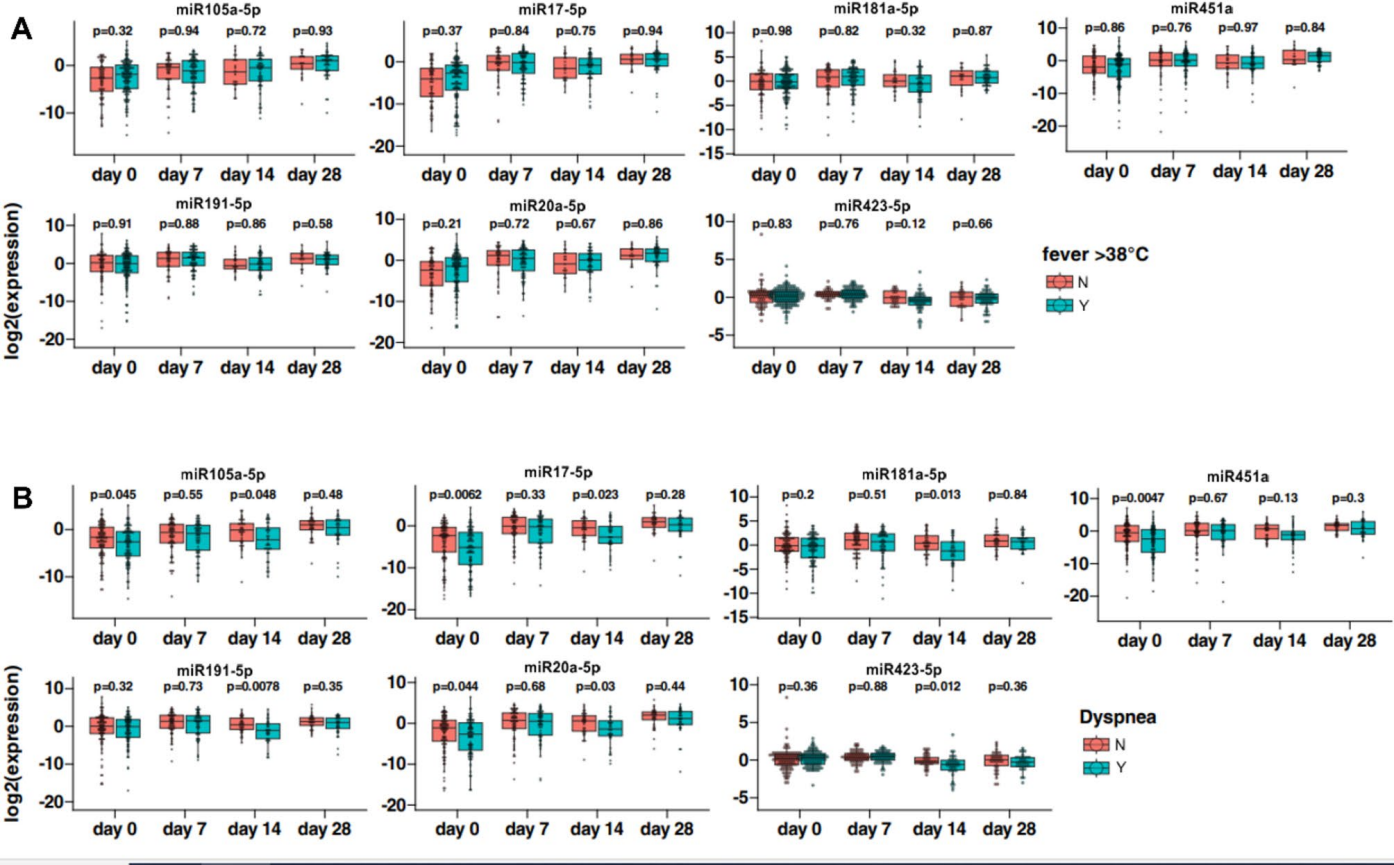
Association of fever (**A**), dyspnea (**B**) with the miRNA expression profile. Mann−Whitney U test, *p* < 0.05 was considered statistically significant. *Y* yes, *N* no.

Fever, is a common symptom of respiratory infections^16^. Fever in COVID-19 patients, especially those requiring ICU admission, is associated with increased mortality^17^. Nonetheless, our results indicate that miRNAs associated with admission to ICU did not differ between individuals with or without fever (Fig. 3A).

In addition, miRNAs miR106a-5p, miR17-5p, miR451a and miR20a had significantly lower serum levels in patients with dyspnea on day 0 and day 14. No other differences were detected (Fig. 3B).

**Fig. 4:**
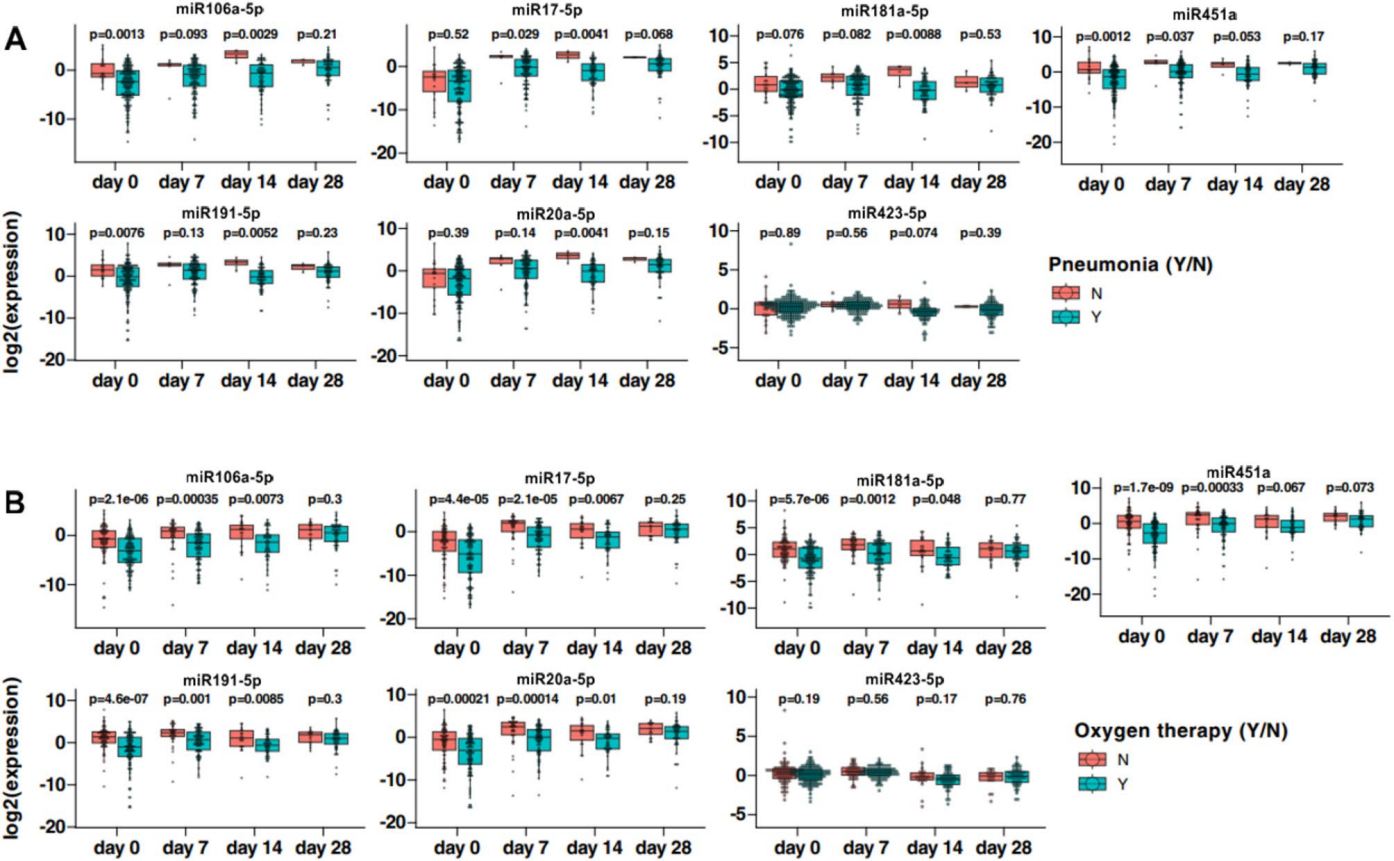
Association of pneumonia (**A**), oxygen therapy (**B**) with the miRNA expression profile. Mann–Whitney U test, *p* < 0.05 was considered statistically significant. *Y* yes, *N* no.

**Fig. 5:**
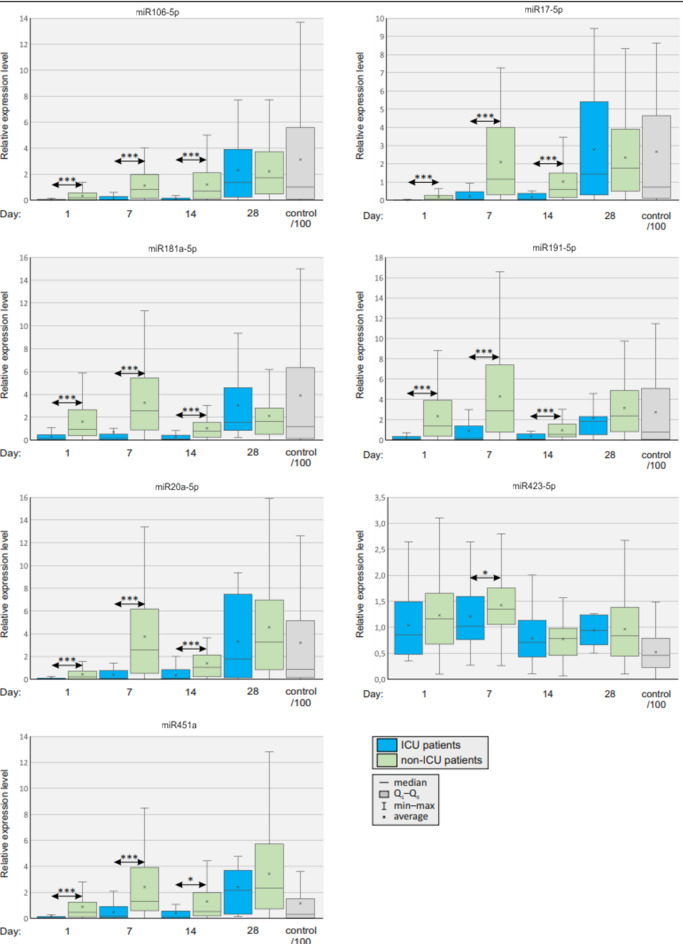
Comparison of miRNA expression profile between ICU and non-ICU patients. Mann–Whitney U test, *p* < 0.05 was considered statistically significant. *p* < 0.05*, *p* < 0.001**, *p* < 0.0001***.

Pneumonia (Fig. 4A) is associated with a global decrease in plasma concentrations of miR106a-5p, miR451a and miR191-5p miRNAs, particularly at day 0. These results are associated with statistically significant lower plasma concentrations of above-mentioned miRNAs in the ICU group (Fig. 5). Simultaneously, it should be noted that there were no statistically significant differences in miR423-5p plasma concentrations between COVID-19 individuals with and without pneumonia, which corresponds to the plasma concentrations of this miRNA in the ICU and non-ICU groups.

Similar phenomena could be observed in the demand for oxygen supply (Fig. 4B). Individuals requiring oxygen therapy had significantly lower plasma levels of all analyzed miRNAs except miR423-5p. As with pneumonia, no differences were found in plasma concentrations of the latter between patients requiring oxygen therapy and those who did not.

## Discussion

The pathophysiology of severe COVID-19 is relatively well established. For instance, a hyperinflammatory response, frequently referred to as the cytokine storm, plays a key role in the unfavorable clinical course of the disease^18^. Nevertheless, there are relatively underexplored aspects that require more in-depth studies. In this paper, we focused on epigenetic mechanisms. More precisely, we investigated the impact of miRNA expression profile upon the course of SARS-CoV-2 infection. MiRNA plays a crucial role in the regulation of gene expression. Typically, it controls mRNA expression, thus acts at the post-transcriptional level^19^. Recent evidence suggests that miRNA also interferes with other non-coding RNAs, such as long non-coding RNAs (lncRNAs), circular RNAs (circRNA), implying that its role in regulating gene expression is more complex than initially assumed^19,20^.

In the first place, the conducted analysis revealed global downregulation of miRNAs in COVID-19 patients compared to healthy controls, which in turn may result in an increase in gene expression, presumably genes promoting the inflammatory response. Alterations in miRNA expression were most prominent at the onset of the disease. The most visible changes were detected at day 0, when 133 differentially expressed miRNAs were identified, and 97 of them were downregulated (Fig. 1). MiRNA clustering and alterations in its expression profile have already been reported in the comparison between severely and moderately ill COVID-19 patients and between healthy controls and with SARS-CoV-2 infected individuals^9^, suggesting its predictive value. Moreover, study revealed the downregulation of a cluster of miRNAs located on chromosome 14 (14q32) among all COVID patients, which among others regulate inflammatory response^9^, what is coherent with the results presented in this study.

Having determined changes in the miRNA expression profile between COVID-19 patients and healthy controls, we aimed to identify differentially expressed miRNAs influencing disease severity, make associations with them and clinical symptoms, and evaluate their prognostic value. We identified miR106a-5p, miR17-5p, miR181a-5p, miR191-5p, miR20a-5p, miR423-5p and miR451a as the differentially expressed miRNAs with highest fold change in expression. The overall trend in the miRNA expression profile clearly demonstrated downregulation of selected molecules in the ICU patients compared to their counterparts with mild to moderate disease. Subsequent analysis showed that plasma concentrations of miRNAs miR106a-5p, miR17-5p, miR181a-5p, miR191-5p, miR20a-5p and miR451a were significantly lower in the ICU group on days 0, 7 and 14. Whereas there were no statistically significant differences in their expression on day 28. Furthermore, in both groups, miRNA expression tends to increase over time being particularly visible on day 28. The latter strongly implies that normalized miRNA expression could be considered a hallmark of recovery. The expression of miRNA miR423-5p was significantly higher in the non-ICU group only at day 7, what suggests that this miRNA is not highly associated with the admission to ICU. Similar to the comparison of COVID-19 and healthy controls, the overall downregulation of miRNAs may result in increased expression of genes, presumably genes promoting the inflammatory response. This may result in a hyperinflammatory response associated with an adverse disease outcome.

There is a shortage of studies investigating the role of miRNA expression profile in the pathobiology of COVID-19 and its predictive value for disease severity. Nevertheless, there are several miRNAs for which evidence is emerging regarding their role in SARS-CoV-2 infection. Our results showed that h20a-5p was significantly downregulated in the ICU patients compared to both the non-ICU group and healthy controls. Li et al. and Hatem et al. demonstrated it to be downregulated in COVID-19 compared to SARS-CoV-2 negative controls^21,22^ whilst Gianella and co-workers revealed its diminished expression in COVID-19, and additionally showed the connection with the disease severity obtaining similar results to ours^23^. Therefore, it can be concluded that miR20a-5p positively predicts both SARS-CoV-2 seropositivity and disease severity. Similar observations were made regarding miR106a-5p. Its decreased expression was associated with SARS-CoV-2 infection and can be considered a reliable marker of COVID-19^22^. Nevertheless, there is a literature gap regarding the association between miR106a-5p and the severity of COVID-19, which is filled by the results of this study, which showed a markedly reduced expression of miR106a-5p. Another miRNA whose lower expression is associated with severe disease is miR17-5p, which is believed to be involved in the antiviral response in COVID-19^22^ and in H7N9 influenza virus infection^24^. Nonetheless, it should be noted that in the study by Zhu and colleagues^25^, miR17-5p expression in H7N9 influenza virus infection was significantly higher than in healthy controls. It can be hypothesized that the low expression of miR17-5p in the ICU group could be attributed to the hyperinflammatory response and excessive consumption of antiviral molecules.

MiR181a-5p was demonstrated to mediate the inflammatory response in sepsis and is significantly upregulated in RAW 264.7 macrophages after lipopolysaccharide stimulation^26^. Nevertheless, Su and colleagues showed that miR181a-5p reduces the expression of pro-inflammatory genes, hence it is downregulated in atherosclerotic plaque and plasma of apoE^−^/^−^ mice^27^, suggesting anti-inflammatory properties resulting from its high expression. In our study, miR181a-5p was significantly downregulated in severely ill COVID-19 individuals, suggesting its role in mediating inflammation. However, consistent with the results obtained by Khatami and colleagues, miR181a was elevated in ICU-hospitalized COVID-19 patients compared to non-hospitalized COVID-19 subjects^28^. Therefore, it appears that the role of miR181a-5p in inflammation, with particular emphasis on COVID-19, requires further in-depth research, especially focusing on downstream mechanisms associated with miR181a-5p up and downregulation. Study conducted by Franco et al. revealed that miR191-5p was significantly downregulated in COVID-19 individuals who required hospital admission^29^. Similarly, our results showed that this miRNA was significantly downregulated in the ICU group on days 0, 7 and 14, further implying its role in mediating unfavorable clinical course of the disease. Moreover, its diminished expression was associated with pneumonia and oxygen therapy in our cohort. The role of miR191-5p in diseases is ambiguous. On the one hand, its low expression contributes, except for the severe course of COVID-19, to other conditions, such as vascular complications in type 1 diabetes^30^. On the other hand, upregulation of this molecules was detected in cancer tissues^31,32^. We also observed low plasma level of miR451a in the ICU group. Moreover, its low expression was associated with the clinical manifestation of the disease. According to the study conducted by Yang et al., low expression of miR451a is associated with the cytokine storm in COVID-19^33^. It should be noted that this miRNA negatively regulates IL-6 expression^34^.

MiRNA is very-well established regulator of gene expression. The above results demonstrated that alterations in miRNA expression might affect clinical course of COVID-19 infection and admission to ICU, presumably because, various inflammatory mediators might be aberrantly expressed. To be precise, downregulation of miRNAs in the ICU group may result in the overexpression of various pro-inflammatory factors. In the aftermath cytokine storm might ensue what clinically manifests itself as a severe disease with non-favorable outcomes.

##  Study limitations

Although our study provided interesting results and novel insights into the pathophysiology of severe COVID-19, it has several drawbacks. First, we focused exclusively on miRNAs. We did not investigate if other epigenetic mechanisms, such as changes in methylation profile, contribute to the severe disease. Changes in methylome that are certainly worth exploring and may additionally reveal novel mechanisms underlying disease severity. Furthermore, the potential downstream mechanisms elicited by alterations in the miRNA expression profile were not further investigated. This issue, although scientifically valuable, is complex and therefore reaches far beyond the scope of this study. Lastly, study subjects were enrolled to the study on the day of hospital admission, not on the first day of illness or onset of clinical symptoms. Nevertheless, they were followed according to the study protocol, and the relatively large sample size ensures the accuracy of the results obtained.

### Conclusion

In summary, the miRNA expression pattern has the potential to predict the severity of COVID-19, reflecting the clinical symptoms of the infection, such as the need for oxygen therapy and concomitant pneumonia. In particular, low expression of miRNAs miR106a-5p, miR17-5p, miR181a-5p, miR191-5p, miR20a-5p and miR451a, especially in the initial phase of the disease, is associated with an unfavorable clinical course of SARS-CoV-2 infection.

## Data Availability

Data are available in the Figshare repository under the URL: 10.6084/m9.figshare.27633885.
